# Phylogenetic relationships of the woodlouse flies (Diptera: Rhinophorinae) and the cluster flies (Diptera: Polleniidae)

**DOI:** 10.1371/journal.pone.0285855

**Published:** 2023-09-19

**Authors:** Silvia Gisondi, Eliana Buenaventura, Arn Rytter Jensen, John O. Stireman, Silvio S. Nihei, Thomas Pape, Pierfilippo Cerretti

**Affiliations:** 1 Department of Biology and Biotechnologies ‘Charles Darwin’, Sapienza University of Rome, Rome, Italy; 2 Natural History Museum of Denmark, Copenhagen, Denmark; 3 Grupo de Entomología Universidad de Antioquia – GEUA, Universidad de Antioquia UdeA, Medellín, Colombia; 4 Department of Biological Sciences, Wright State University, Dayton, Ohio, United States of America; 5 Department of Zoology, Institute of Biosciences, University of São Paulo, São Paulo, Brazil; 6 Museo di Zoologia, Polo Museale Sapienza, Sapienza University of Rome, Rome, Italy; Nanjing Agricultural University, CHINA

## Abstract

Phylogenetic relationships within the oestroid subclades Rhinophorinae (Calliphoridae) and Polleniidae were reconstructed for the first time, applying a Sanger sequencing approach using the two protein-coding nuclear markers CAD (carbamoyl-phosphate synthetase 2, aspartate transcarbamylase, and dihydroorotase; 1794 bp) and MCS (molybdenum cofactor sulfurase; 2078 bp). Three genera of Polleniidae and nineteen genera of Rhinophorinae were analyzed together with a selection of taxa representing the major lineages of Oestroidea (non-rhinophorine Calliphoridae, Oestridae, Sarcophagidae, Tachinidae). The selected markers provide good resolution and moderate to strong support of the distal branches, but weak support for several deeper nodes. Polleniidae (cluster flies) emerge as monophyletic and their sister-group relationship to Tachinidae is confirmed. *Morinia* Robineau-Desvoidy as currently circumscribed emerges as paraphyletic with regard to *Melanodexia* Williston, and *Pollenia* Robineau-Desvoidy is the sister taxon of the *Morinia*–*Melanodexia* clade. We propose a classification with two subfamilies, Moriniinae Townsend (including *Morinia*, *Melanodexia*, and *Alvamaja* Rognes), and Polleniinae Brauer & Bergenstamm (including *Pollenia*, *Dexopollenia* Townsend, and *Xanthotryxus* Aldrich). *Anthracomyza* Malloch and *Nesodexia* Villeneuve are considered as Oestroidea *incertae sedis* pending further study. Rhinophorinae (woodlouse flies) emerge as monophyletic and sister to a clade composed of (Ameniinae + (Ameniinae + Phumosiinae)), and a tribal classification is proposed with the subfamily divided into Rhinophorini Robineau-Desvoidy, 1863 and Phytonini Robineau-Desvoidy, 1863 (the *Stevenia*-group and the *Phyto*-group of authors, respectively). *Oxytachina* Brauer & Bergenstamm, 1891, **stat. rev.** is resurrected to contain nine Afrotropical rhinophorine species currently assigned to genus *Rhinomorinia* Brauer & Bergenstamm, 1891: *Oxytachina approximata* (Crosskey, 1977) **comb. nov.**, *O*. *atra* (Bischof, 1904) **comb. nov.**, *O*. *bisetosa* (Crosskey, 1977) **comb. nov.**, *O*. *capensis* (Brauer & Bergenstamm, 1893) **comb. nov.**, *O*. *scutellata* (Crosskey, 1977) **comb. nov.**, *O*. *setitibia* (Crosskey, 1977) **comb. nov.**, *O*. *verticalis* (Crosskey, 1977) **comb. nov.**, *O*. *vittata* Brauer & Bergenstamm, 1891, and *O*. *xanthocephala* (Bezzi, 1908) **comb. nov.**

## Introduction

Oestroidea comprise a diverse clade of true flies comprising some of the most familiar insects, such as blow flies and flesh flies. The group accounts for about 15,000 known species [1], but estimates suggest that the true number may be at least twice as many [2, 3]. As holometabolous insects, their larval stage is morphologically and functionally entirely different from the adult stage, and whereas adults are often flower visitors, oestroid larvae can be general scavengers; vertebrate or invertebrate necrophages; vertebrate coprophages; vertebrate parasites; invertebrate parasitoids; predators of frog spawn, molluscs, earthworms, termites, grasshopper eggs or spider eggs; and even mycophages and palynophages [2, 4, 5].

Reconstructing the phylogenetic relationships among oestroid lineages has long represented a major challenge. Morphology has provided sparse and conflicting evidence [6, 7], and molecular studies have differed, mainly relating to gene-choice and taxon sampling [8–10]. However, consensus on the oestroid backbone is now emerging through phylogenomic and phylotranscriptomic approaches [11–13]. In-depth phylogenetic studies aiming at the reconstruction of relationships within particular clades have also been performed [13 (Calliphoridae); 14, 15 (Oestridae); 12, 16 (Sarcophagidae); 17 (Mesembrinellidae); 18 (Tachinidae)]. The present paper focuses on two oestroid subclades for which molecular genus-level phylogenies are still largely lacking, namely the Rhinophorinae and the Polleniidae.

**Fig 1:**
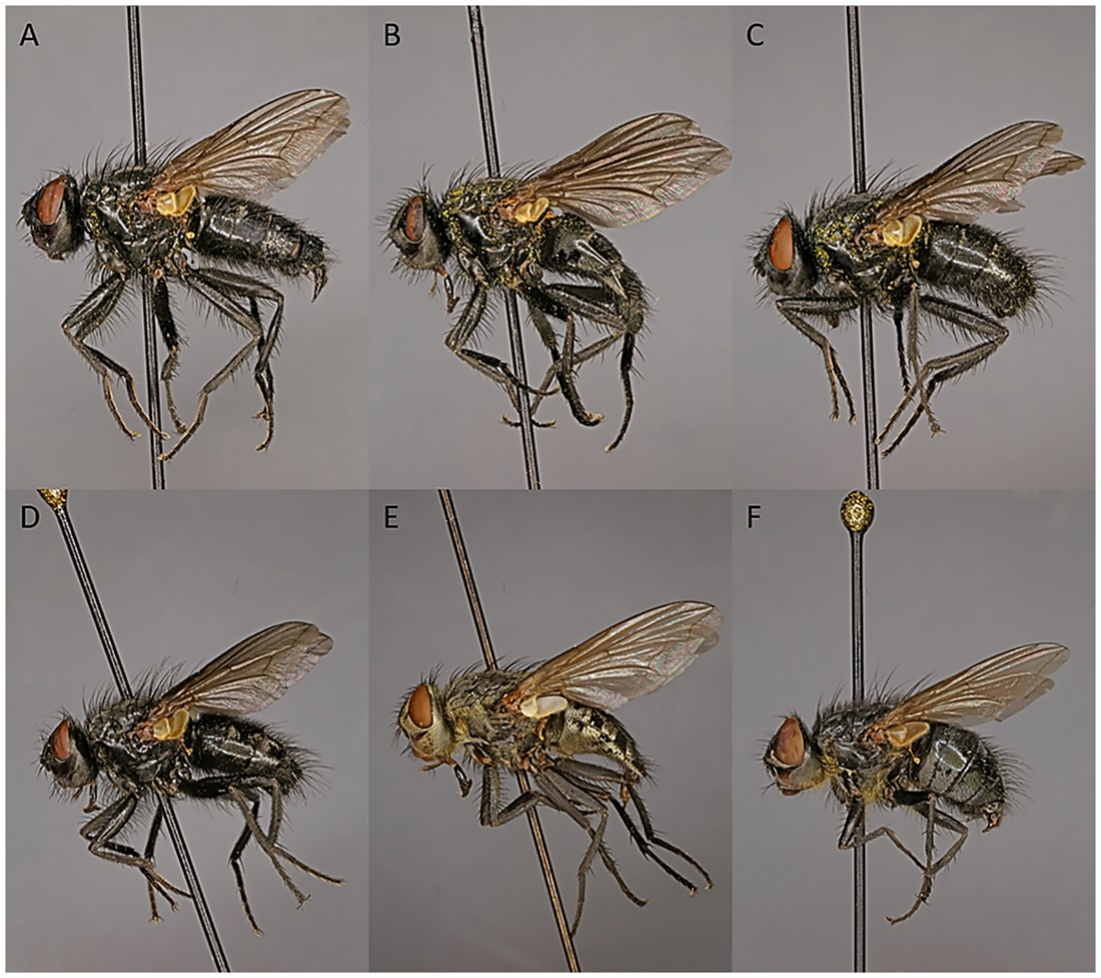
Polleniidae species included in the present analyses; adult habitus. **A** *Melanodexia glabricula* (Bigot, 1887). **B** *Melanodexia grandis* (Shannon, 1926). **C** *Melanodexia tristina* (Hall, 1948). **D** *Melanodexia tristis* Williston, 1893. **E** *Pollenia rudis* (Fabricius, 1794). **F** *Pollenia* nr. *stolida* Malloch. [A–D, F = male; E = female].

Polleniidae (Fig 1)—also known as cluster flies due to the tendency of adults of some species to cluster indoors for overwintering—is a family of earthworm parasitoids [19–23]. The polleniids have been treated either as a subfamily (Polleniinae) or tribe (Polleniini) within the Calliphoridae [24–27] or given family rank [28]. Polleniids have recently become well-established as the extant sister taxon of the megadiverse parasitoid clade Tachinidae [11, 12, 18, 29–33], even though morphology or other character systems so far have provided few clues in support of this relationship. Studies of the phylogenetic relationships among polleniid genera are limited and include only sparse taxon sampling [28, 34, 35]. Currently the family contains some 150 named species in eight genera, with the bulk of diversity in the Palaearctic Region [36]. Polleniids are also widespread and abundant in the Oriental and Australasian regions, but native species are confined to smaller areas in the Nearctic (West Coast of the USA) and the Afrotropics (southern Africa), and are entirely absent from the Neotropics [36, 37]. A few species have become widely distributed, possibly due to individuals diapausing in shipping containers and with the widespread establishment of introduced populations of their host earthworms [38].

**Fig 2:**
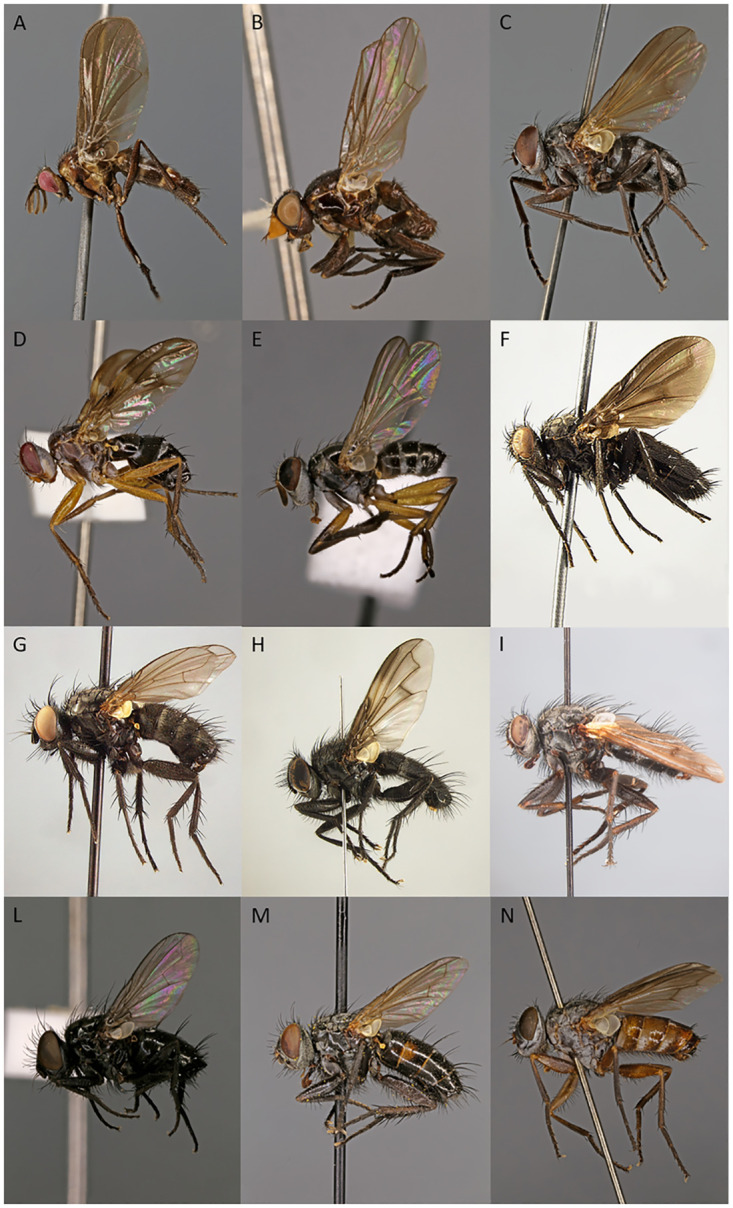
Rhinophorinae species included in the present analyses; adult habitus. **A** *Aporeomyia elaphocera* Gisondi, Pape, Shima & Cerretti, 2020. **B** *Axinia arenaria* Colless, 1994. **C** *Baniassa pennata* Gisondi, Pape, Shima & Cerretti, 2020. **D** *Bezzimyia yepezi* Pape & Arnaud, 2001. **E** *Bixinia winkleri* Cerretti, Lo Giudice & Pape, 2014. **F** *Melanophora roralis* (Linnaeus, 1758). **G** *Oplisa tergestina* (Schiner, 1861). **H** *Paykullia partenopea* (Rondani, 1861). **I** *Paykullia* nr. *nubilipennis*. **L** *Rhinophora lepida* (Meigen, 1824). **M** *Stevenia deceptoria* (Loew, 1847). **N** *Tromodesia angustifrons* Kugler, 1978. [A–N = male].

Rhinophorinae (Calliphoridae) (Fig 2) is a small clade of woodlouse parasitoids, which, as for the cluster flies, have also been bouncing around within the Oestroidea. The group was long considered a subfamily, either under Calliphoridae [22, 39–42] or Tachinidae [43–48], or treated at family rank [6, 7, 12, 49–61]. Recently, however, Yan et al. [13], based on transcriptomes and a limited taxon sampling, reclassified part of the oestroids proposing woodlouse flies as a subfamily of Calliphoridae with a sister-group relationship to the Ameniinae (including the Helicoboscinae) [11, 13]. Regardless of taxonomic ranking, the monophyly of woodlouse flies has never been questioned, although the lack of unique adult synapomorphies and only scattered information on immature stages and natural history has caused several genera to shift either into or out of the taxon [61]. At present, rhinophorines number 180 species in 33 genera, and their species diversity peaks in the Mediterranean area [61, 62]. Woodlouse flies are widespread except for a notable absence from temperate North America, where they are only represented by a few species recently introduced from the Palaearctic Region [63, 64]. This peculiar distribution may be due to the paucity of native Nearctic terrestrial isopod species [65–68], or to low host population densities [69–71].

The present paper provides a comprehensive phylogeny of the Rhinophorinae and the Polleniidae, involving an extensive taxon sampling and employing two nuclear protein-coding genes, CAD and MCS, previously evaluated as having high phylogenetic informativeness [31, 72].

## Materials and methods

Ethanol-preserved material was obtained for Polleniidae (3 out of 8 currently recognized genera) and Rhinophorinae (19 out of 33 currently recognized genera), with a complete biogeographical coverage, and a set of outgroup taxa (49 genera) representing Calliphoridae (Ameniinae, Bengaliinae, Calliphorinae [including calliphorine taxa formerly in Melanomyinae and Toxotarsinae], Chrysomyinae, Luciliinae, Phumosiinae), Mesembrinellidae, Oestridae, Sarcophagidae, Tachinidae (Dexiinae, Exoristinae, Phasiinae, Tachininae) and Ulurumyiidae (see S1 Table). GenBank sequences were included from Winkler et al. [31] and for *Musca domestica* Linnaeus for outgroup rooting. Extractions and amplifications were carried out at the GeoGenetics Lab at University of Copenhagen, Denmark, while sequencing was outsourced to Macrogen Europe (Amsterdam, the Netherlands).

CAD (carbamoyl-phosphate synthetase 2, aspartate transcarbamylase, and dihydroorotase) and MCS (molybdenum cofactor sulfurase) were chosen for their phylogenetic information and reliability for Mesozoic-Cenozoic-aged explosively radiated groups such as the Oestroidea [72], as well as for ease of comparisons with results and integration of sequences from analyses of other available datasets.

Three legs were removed from ethanol-preserved specimens and stored in ethanol until extraction. Extractions were performed using the DNeasy Blood and Tissue Kit (Quiagen, Venlo, the Netherlands) with the following modifications of the manufacturer’s protocol: legs were placed entire in the digestion buffer and Buffer ATL was replaced with a digestion buffer as described by Gilbert et al. [73] but modified to consist of 10 mM Tris-HCl (pH 8), 10 mM NaCl, 5 mM CaCl_2_, 2.5 mM EDTA, 1% sodium dodecyl sulphate (SDS), 250 μg/mL proteinase K, and 40 mM dithiotreitol (DTT) (final concentrations).

PCR amplification reactions (total volume 25 μL) were composed of 18 μL deionized water, 4 μL of 5X HOT FIREPol Blend Master Mix (Solis BioDyne), 0.5 μL of each primer (final concentration of 0.2 μM), and 2 μL of DNA solution. The most effective program among all the experimental variations was a PCR protocol consisting of an initial denaturation stage of 12 min at 95°C; 35 cycles of 95°C for 30 sec, variable annealing temperature (depending on the primers used, see Table 1) for 1 min, 72°C for 2 min, and a final extension time of 10 min at 72°C. After visualization on a 2% Agarose gel, PCR products were sent to Macrogen Europe (Amsterdam, the Netherlands) for PCR product cleanup and sequencing.

**Table 1:** Primers used and their annealing temperatures.

Name	Direction	Sequence 5’-3’	Nucleotides	Annealing Temperature
Rhino_CAD6_f	Forward	CATTTGGAGTGGTTGGAAGG	20	49 °C
Rhino_CAD4_r	Reverse	GACAACAACTGATGACCTAAAC	22	
Rhino_CAD5_f	Forward	CGTAATTTGGTGGCCGAGTG	20	49 °C
Rhino_CAD7_r	Reverse	CCAAAAGTCAATAGCACCCC	20	
Rhino_MCS8_f	Forward	GCTACTGCGGCCTTAAAAAC	20	50 °C
Rhino_MCS3_r	Reverse	CCCGAACATTTTGTAGAATG	20	
Rhino_MCS1_f	Forward	GCTCAATGTAATTTTAGTGG	20	41–48 °C
Rhino_MCS2_r	Reverse	ACAATTAAAGCACCTACTCC	20	

Sequencing output files were assembled and trimmed using Geneious 9.1.8 (Biomatters Ltd., Auckland, New Zealand). FASTA files of sequences were aligned using MAFFT (v.7.017) with the G-INS-i algorithm using the default parameters [74, 75] (see S1 Dataset). The resulting alignments were checked for accuracy by looking for stop codons and spurious gaps once the alignments were translated into proteins. The single-gene alignments were then concatenated using the “Concatenate alignments” tool in Geneious (see S1 Dataset).

PartitionFinder v2 [76, 77] was used to find the best-fitting partitioning scheme and to select substitution models for each partition without overparameterization, evaluated by the information-theoretic metric BIC (Bayesian Information Criterion). The initial 6 data blocks were the first, second, and third codon positions of each of CAD and MCS, and the program was set to perform a greedy search to compare all possible partitioning schemes. The best-fit scheme grouped all data blocks in one single partition. The model GTR+I+G was selected as the substitution model for this partition.

Likelihood analyses were conducted using RAxML version 8.2.12 [78] on XSEDE (Extreme Science and Engineering Discovery Environment) through the CIPRES (Cyberinfrastructure for Phylogenetic Research) Science Gateway [79]. The tree of highest likelihood from 100 replicate runs was selected for plotting the bootstrap values from 250 ML rapid bootstrap replicates obtained through a GTR+G+I approximation. Trees from all analyses were visualized using FigTree [80].

## Results

**Fig 3:**
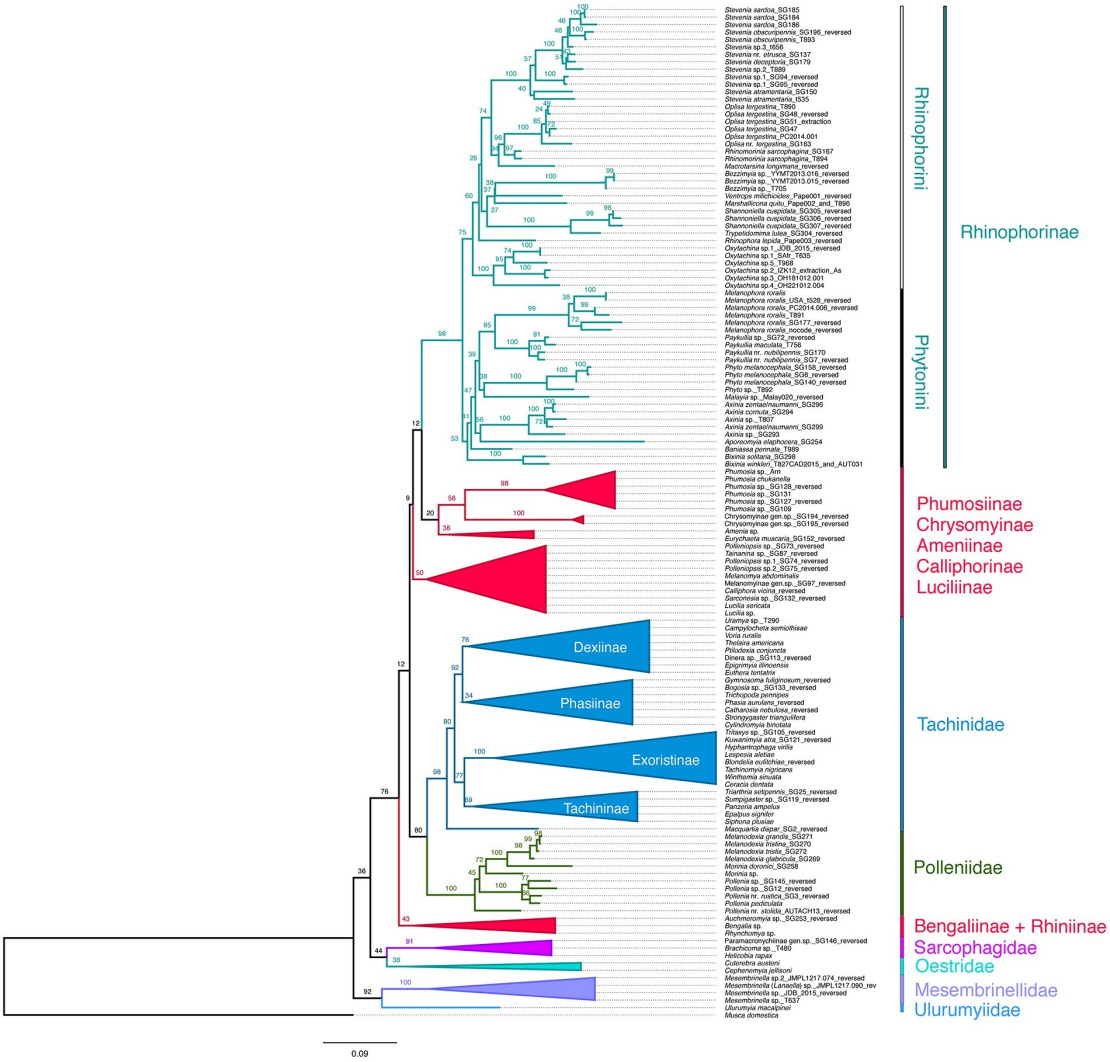
ML tree with bootstrap values mapped.

Analysis of the concatenated matrix from CAD (1794 bp) and MCS (2078 bp) resulted in a well-resolved ML topology, although with some branches having low bootstrap support values (henceforth b.v.) (Fig 3).

The ML tree (Fig 3) shows overall strong support for shallow nodes within families, but somewhat lower support for many of the deeper branches representing relationships among families and other major clades. Relationships among the genera within the target cluster flies and woodlouse flies are generally robust and well-resolved. DNA sequence data for *Aporeomyia* Pape & Shima, *Baniassa* Kugler, *Malayia* Malloch, *Macrotarsina longimana* (Egger), *Marshallicona* Cerretti & Pape, *Shannoniella* Townsend, *Trypetidomima* Townsend and *Melanodexia* Williston are here obtained for the first time.

The recently recircumscribed Calliphoridae are divided into a grade of four clades as follows: Bengaliinae (including Auchmeromyiini and Bengaliini) + Rhiniinae (b.v. = 43); Luciliinae + Calliphorinae (b.v. = 50); and Ameniinae + (Chrysomyinae + Phumosiinae) (b.v. = 20). Our analysis reconstructed the clade Tachinidae + Polleniidae (b.v. = 80) nested within the calliphorid grade and sister to the core Calliphoridae (i.e., excluding the Bengaliinae + Rhiniinae clade), but overall statistical support is low. The sister group to Rhinophorinae is a clade composed of [Ameniinae (b.v. = 38) + [Chrysomyinae (b.v. = 100) + Phumosiinae (b.v. = 98)]]; however, these relationships are only weakly supported.

Sarcophagidae form a low-supported clade (b.v. = 44), which is sister taxon to a low-supported Oestridae (b.v. = 38), while Mesembrinellidae are retrieved as well supported (b.v. = 100) monophyletic group sister to Ulurumyiidae (b.v. = 92).

Our analyses recover a monophyletic Polleniidae with strong support (b.v. = 100). Within this clade, *Pollenia* nr. *stolida* Malloch from Australia is reconstructed as sister to the remaining polleniids; however, the latter clade has low support (b.v. = 45). The remaining species of *Pollenia* Robineau-Desvoidy form a well-supported clade (b.v. = 100), sister to the *Melanodexia*–*Morinia* clade composed of a monophyletic *Melanodexia* (b.v. = 98) reconstructed as sister to *Morinia doronici* (Scopoli), and with a subordinate *Morinia* sp. from South African rendering *Morinia* Robineau-Desvoidy paraphyletic.

Rhinophorinae are retrieved as monophyletic (b.v. = 98) as are the two subgroups, each of which is characterized by a highly derived larval morphology and locomotory behaviour (see further below), i.e., Phytonini (b.v. = 53) and Rhinophorini (b.v. = 75). Among the included non-monotypic genera for which we included more than one species, *Axinia* Colless, *Bixinia* Cerretti, Lo Giudice & Pape, *Paykullia* Robineau-Desvoidy, *Phyto* Robineau-Desvoidy and *Stevenia* Robineau-Desvoidy emerged as monophyletic with strong support (b.v. = 100), whereas the species traditionally assigned to genus *Rhinomorinia* Brauer & Bergenstamm separated into two geographically disjunct groups: an Afrotropical clade (henceforth as *Oxytachina* Brauer & Bergenstamm **stat. rev.,** b.v. = 100) and a Palaearctic clade (*Rhinomorinia*), both belonging to the Rhinophorini. *Oxytachina* is reconstructed as sister to the remaining Rhinophorini. Within this tribe, the Afrotropical genus *Ventrops* Crosskey is reconstructed in a nested position within a clade of Neotropical endemic taxa (*Bezzimyia* Townsend, *Marshallicona* Cerretti & Pape, *Shannoniella* Townsend and *Trypetidomima* Townsend), but overall support is weak. The clade composed of the Palaearctic *Macrotarsina* Schiner, *Rhinomorinia*, *Oplisa* Rondani and *Stevenia* received moderate support (b.v. = 74). Within the Phytonini, deeper branches have low or moderate support (b.v. = 38–85). *Bixinia* spp. emerge as sister to the remaining Phytonini, with *Baniassa pennata* Gisondi, Pape, Shima & Cerretti as the next most basal branching. Sister to *Baniassa* is a weakly supported clade (b.v. = 47) composed of the Australasian *Aporeomyia* + *Axinia* clade (b.v. = 56) and the Oriental/Palaearctic [*Malaya* + *Phyto*] (b.v. = 38) + [*Paykullia* + *Melanophora*] (b.v. = 85) clade (b.v. = 39).

## Discussion

A fully resolved and well supported phylogeny of oestroid flies has proved difficult to attain through both morphological and Sanger-generated molecular data. However, a consensus on the topology of the backbone is now emerging through phylogenomic and phylotranscriptomic approaches [11–13]. Conflicts in the deeper splits, i.e., in the position of families and subfamilies between the present study and the more recent phylogenomic studies are here considered as most likely resulting from our use of data from only two nuclear loci. Many deep nodes received low statistical support values, and they are not discussed further.

Despite recent study, the phylogenetic relationships among polleniid genera are still tentative. Employing a combination of morphological characters and fragments of three nuclear markers (CAD, MCS, MAC) on a selection of *Morinia* species, one *Pollenia* and the monotypic *Alvamaja* Rognes, Cerretti et al. [28], reconstructed *Morinia* as monophyletic and sister to *Alvamaja*, this clade being sister to *Pollenia*. Recently, Johnston et al. [35] presented a mitogenomic analysis of 21 polleniid taxa, including a broad representation of West Palaearctic *Pollenia* and one species each of *Melanodexia*, *Morinia* and *Dexopollenia* Townsend. The study retrieved *Dexopollenia* as sister to a clade composed of *Morinia* and *Melanodexia*, with this clade in turn sister to *Pollenia*. Johnston et al. [35] performed further analyses by using COI sequences (i.e., not the entire mitogenome) of *Xanthotryxus mongol* Aldrich and an additional species of *Morinia*, and recovered *Dexopollenia* + *Xanthotryxus* Aldrich as sister to the remaining Polleniidae, and the latter resolving as *Pollenia* being sister to *Morinia* + *Melanodexia*. These relationships come with low support, which is partly obscured by Johnston et al. [35] incorrectly using support values for particular nodes to indicate support for the basal dichotomy. However, the present ML topology is largely consistent with the results in Johnston et al. [35] (except for retrieving *Pollenia* as paraphyletic), but the limited taxon sampling does not allow for testing the phylogenetic position of *Dexopollenia* and *Xanthotryxus*. The sparse morphological evidence tends to support a *Dexopollenia*–*Xanthotryxus*–*Pollenia*-clade. All species of *Xanthotryxus* and most species of *Dexopollenia* and *Pollenia* share the presence of golden, wavy, hair-like setae on parts of the body, and the morphologically very similar *Pollenia* and *Xanthotryxus* also share a subcostal sclerite with a bundle of long, black or yellow setae among the micropubescence. However, comparative morphology of polleniids needs much more study, and differing phylogenetic topologies obscure interpretations of character state polarities. For instance, the Australian *Pollenia* nr. *stolida* examined here differs from the Palaearctic and New Zealand species of *Pollenia* by lacking the first presutural intra-alar seta and by the three preapical setae of the hind tibia (anterodorsal, dorsal and posterodorsal) being subequal in size; both these character states are shared with *Morinia* and *Melanodexia* and may represent plesiomorphic conditions. Interestingly, we found this species taking up the position of sister taxon to all other polleniids included in the analyses, although with weak support (Fig 3).

The *Morinia*–*Melanodexia* clade is supported by the following putative morphological autapomorphies: i) narrow, tongue-shaped, lower calypter, ii) posterior spiracle with reduced posterior lappet (rhinophorine-like) and iii) node at base of R_4+5_ bare. *Morinia* is here represented by the type species *M*. *doronici* (Scopoli) (Palaearctic) and by an undescribed species from South Africa [19, 29]. Our phylogeny reconstructed the two included species of *Morinia* as paraphyletic with respect to *Melanodexia*. Indeed, our careful examination of *Morinia* from both Palaearctic and Afrotropical regions has not revealed any strong evidence supporting their monophyly, except for sharing a slim, narrow, body shape, which contrasts with the stouter body characterizing the other polleniids, except *Alvamaja*. Despite this, we consider it premature to lump species currently assigned to *Morinia* and *Melanodexia* under the same genus-group name as long as there is inconclusive data on the phylogenetic position of *Alvamaja*, *Anthracomyza* Malloch, *Dexopollenia* Townsend, *Nesodexia* Villeneuve and *Xanthotryxus*, all of which may belong in the Polleniidae [36]. *Alvamaja* presents a unique combination of character states and could belong to the *Morinia*–*Melanodexia* clade based on its rhinophorine-like (i.e., non-operculate) posterior spiracle and narrow lower calypter. This relationship is supported by the morphological evidence presented in Cerretti et al. [28]. *Dexopollenia* and *Xanthotryxus* share several, derived character states with *Pollenia*, including the golden, wavy hair-like setae particularly abundant on the thorax, and the cluster of long black or yellow setae on the subcostal sclerite. No progress has been made so far in resolving the phylogenetic placement of *Anthracomyza* and *Nesodexia*. These are both monotypic genera and no molecular sequence data have been obtained from them. Although several polleniid nominal genus-group taxa remain to be included in a phylogenetic analysis, we are here proposing to apply a subfamily classification with the Polleniidae composed of two subfamilies, as follows:

**Moriniinae** Townsend: including *Morinia*, *Melanodexia*, and *Alvamaja*;
**Polleniinae** Brauer & Bergenstamm: including *Pollenia*, *Dexopollenia*, and *Xanthotryxus*.

We here treat *Anthracomyza* and *Nesodexia* as Oestroidea *incertae sedis* pending further study.

Our study is the first attempt at resolving the phylogenetic relationships within the woodlouse flies using molecular data. Analyses support both the monophyly of the subfamily and its division into two subclades, which we propose here as tribes: Rhinophorini and Phytonini (i.e., the *Stevenia* group and the *Phyto* group of Pape & Arnaud [81], respectively). By integrating our results (Fig 3) with those from previous phylogenetic reconstructions, deduced from morphological data and a larger taxon sampling [61, and literature therein], the two recognized tribes are composed as follows (an asterisk indicates taxa which have not been placed based on molecular data):

**Rhinophorini:** *Acompomintho* Townsend*, *Apomorphyto* Cerretti, Lo Giudice & Pape*, *Azaisia* Villeneuve*, *Bezzimyia*, *Macrotarsina*, *Maurinophora* Cerretti & Pape*, *Melanomyiodes* Crosskey*, *Marshallicona*, *Metoplisa* Kugler*, *Neotarsina* Cerretti & Pape*, *Oplisa*, *Oxytachina* **stat. rev.**, *Queximyia* Crosskey*, *Rhinomorinia*, *Rhinophora* Robineau-Desvoidy, *Shannoniella*, *Stevenia*, *Tricogena* Rondani*, *Tromodesia* Rondani*, *Trypetidomima*, *Ventrops*;
**Phytonini:** *Aporeomyia*, *Axinia*, *Baniassa*, *Bixinia*, *Comoromyia* Crosskey*, *Kinabalumyia* Cerretti & Pape*, *Malayia*, *Melanophora* Meigen, *Parazamimus* Verbeke*, *Paykullia*, *Phyto*, *Rhinodonia* Cerretti, Lo Giudice & Pape* and *Rhinopeza* Cerretti, Lo Giudice & Pape*.

Within Rhinophorini, all the included non-monotypic genera emerged as monophyletic except for “*Rhinomorinia*”. This nominal genus was retrieved as polyphyletic, being divided into two well-supported lineages, which are characterized by distinctive morphological features [60–62; see also the key below]. One clade comprises exclusively Afrotropical species (here placed in the resurrected nominal genus *Oxytachina* **stat. rev.**, see below) and was retrieved as sister to the remaining Rhinophorini with moderate support. The other clade comprises two Palaearctic/western Oriental species, of which *Rhinomorinia sarcophagina* (Schiner) (type species of the genus) was included and clustered within the Rhinophorini as sister to *Oplisa* with strong support. Under ML, *Rhinophora lepida* (Meigen) was recovered as sister to all Rhinophoriini except *Oxytachina*, differing from the morphology-based phylogeny of Cerretti et al. [61] that retrieved *Rhinophora* joining a primarily Palaearctic subclade composed of *Rhinomorinia*, *Macrotarsina*, *Oplisa* and *Stevenia*, which also contained some Afrotropical and Oriental species for which molecular data are not available. The position of the Afrotropical genus *Ventrops* within an otherwise Neotropical clade is biogeographically challenging, but the low support indicates that this hypothesis needs further testing. Support values for the genus-level reconstruction within this clade were weak to moderate. Interestingly, the morphology-based phylogeny of Cerretti et al. [61] had *Ventrops* as the sister taxon of a clade containing all the Neotropical taxa and the Australian genus *Bixinia*. Analyses of morphological data separated the genus *Bixinia* widely from the other Australasian taxa [61], while the present molecular analysis of *Axinia* and *Bixinia* places these genera within the same tribe but separated by multiple intervening genera (Fig 3).

### Changes in classification

For the Polleniidae, we propose a classification into two subfamilies, Moriniinae Townsend, 1919, **stat. nov.**, and Polleniinae Brauer & Bergenstamm, 1891, **stat. nov.** The genera *Anthracomyza* and *Nesodexia* are considered as Oestroidea *incertae sedis*.

For the Rhinophorinae, we propose:

i) classification into two tribes, Rhinophorini Robineau-Desvoidy 1863, **stat. nov.** and Phytonini Robineau-Desvoidy 1863, **stat. nov.**
ii) resurrection of the genus-group name *Oxytachina* Brauer & Bergenstamm, 1891, **stat. rev.**, to accommodate nine Afrotropical rhinophorine species formerly assigned to genus *Rhinomorinia* [61]: *Oxytachina approximata* (Crosskey, 1977) **comb. nov.**, *O*. *atra* (Bischof, 1904) **comb. nov.**, *O*. *bisetosa* (Crosskey, 1977) **comb. nov.**, *O*. *capensis* (Brauer & Bergenstamm, 1893) **comb. nov.**, *O*. *scutellata* (Crosskey, 1977) **comb. nov.**, *O*. *setitibia* (Crosskey, 1977) **comb. nov.**, *O*. *verticalis* (Crosskey, 1977) **comb. nov.**, *O*. *vittata* Brauer & Bergenstamm, 1891 (type species of the genus) **comb. nov.**, and *O*. *xanthocephala* (Bezzi, 1908) **comb. nov.** The genus *Rhinomorinia* is redefined and now consists of only two Palaearctic species: *R*. *sarcophagina* (type species of the genus) and *R*. *longifacies* Herting, 1966.

The following differential diagnosis helps to separate *Oxytachina* Brauer & Bergenstamm, 1891 from *Rhinomorinia* Brauer & Bergenstamm, 1889:

***Rhinomorinia* Brauer & Bergenstamm, 1889 [Palaearctic Region]:** First postsutural supra-alar seta present and well developed, as long as or longer than notopleural setae. Three anterodorsal setae on mid tibia;
***Oxytachina* Brauer & Bergenstamm, 1891 [Afrotropical Region]:** First postsutural supra-alar seta absent or very short, distinctly shorter and weaker than notopleural setae. One or two anterodorsal setae on mid tibia

## Conclusions

Until recently, the taxonomic boundaries and phylogenetic affinities of Polleniidae and Rhinophorinae–two key groups of parasitoids of soil-dwelling organisms–remained controversial. Our analysis, despite being limited to two protein-coding nuclear genes, confirmed previous hypotheses on the relationships between the two groups and provided new insights into their internal phylogenetic relationships. These results allowed us to formally propose a subfamilial and tribal classification for the polleniids and rhinophorines, respectively, and to resurrect the genus *Oxytachina* to include five Afrotropical species previously assigned to the genus *Rhinomorinia*, which thereby is restricted to two Palaearctic species.

## Supporting information

S1 Table Sampled taxa with voucher number, sampling locality and GenBank accession numbers. (XLSX)

S1 Dataset Nexus file containing the dataset matrix for the phylogenetic analysis. (TXT)
